# Trauma Surgeons’ Perspective on Gun Violence and a Review of the Literature

**DOI:** 10.7759/cureus.3599

**Published:** 2018-11-16

**Authors:** Luke J Hofmann, Natasha Keric, Ramon F Cestero, Rachelle Babbitt-Jonas, Leen Khoury, Melissa Panzo, Javier Martin Perez, Stephen M Cohn

**Affiliations:** 1 Surgery, University of Texas Health Science Center, San Antonio, USA; 2 Surgery, Banner University Medical Center, Phoenix, USA; 3 Surgery, Staten Island University Hospital, Staten Island, USA; 4 Emergency Medicine, Staten Island University Hospital, Staten Island, USA; 5 Surgery, Hackensack Meridian Health, Hackensack, USA

**Keywords:** handgun, firearm, gun violence, gun laws, gun control

## Abstract

Background

In the United States, there is a constant debate between the proponents of the right to bear arms and those desiring to reduce the epidemic of gun violence. We sought to capture the trauma surgeons' perspective on gun control.

Methods

We presented an on-line based survey to the members of the American Association for the Surgery of Trauma (AAST). Survey questions were chosen to reflect the popular media poll questions as well as trauma-specific perspectives. We compared the trauma surgeons' perspectives to that of the general populace from a poll conducted by the New York Times (NYT).

Results

A total of 120 trauma surgeons responded to the survey. The age group ranged from 34 to 82 years, and the median age was 51. Most respondents were male (64%, *n* = 67) and worked at a Level I trauma center (80%, *n *= 96) in an academic setting (67%, *n *= 80). About half of the responding surgeons owned a household firearm (40%; *n *= 48 of the AAST members vs. 47%; *n *= 521 of the general populace). Sixty-one percent of the trauma surgeons (*n *= 73) and 53% (*n *= 588) of the NYT respondents favor stricter gun control laws. While 80% (*n *= 888) of the NYT respondents felt that mental health screening and treatment would decrease gun violence, only 56% (*n* = 67) of surgeons felt that mental health screening would be beneficial. The majority (90%, *n *= 999) of the NYT poll respondents favor a law restricting the sale of guns only by licensed dealers. Only (66%, *n *= 79) of the trauma surgeons were in agreement with the stricter gun sale legislation by licensed dealers.

Conclusion

Trauma surgeons appear to share similar views with the general American populace regarding gun violence and injury control.

## Introduction

Incidents of recent mass-casualty gun violence are engraved deeply in our minds: The High School in Parkland, FL February 2018; The Church in Sutherland Springs, TX November 2017; The Concert in Las Vegas, NV October 2017, The Nightclub in Orlando, FL June 2016; and The Elementary School in Newtown, CT December 14, 2012. Gun violence accounts for 18,000 murders and 35,000 suicides annually [1]. Approximately 67,000 people survive gun injuries annually according to the National Center for Injury Prevention and Control. The homicide rate in the United States is 6.9 times higher than the rates in 22 other high-income countries combined [2]. While the ultimate goal of a trauma surgeon is to provide exceptional care to the victims of gun violence, the most effective way to ensure safety starts with injury prevention.

Prevention of gun-related deaths requires an appreciation of the current issues and government policies surrounding gun violence and their effects on our healthcare system. Gun regulation depends on many factors such as gun ownership, gun control laws (to include legislation, sale, and magazine capacity), impact of popular culture, influence of police and security personnel, and the effect of mental health screening on gun violence. Trauma surgeons, by virtue of their profession, may have a unique perspective on how gun violence impacts public health. This knowledge may guide legislative efforts to improve safety.

## Materials and methods

The Institutional Review Board of the University of Texas Health Science Center at San Antonio approved and provided oversight for this research. Electronic surveys were distributed in July 2013 to the members of the American Association for the Surgery of Trauma (AAST) via a contact list containing members’ email addresses. The email invitation included a link to access the Survey MonkeyÒ site, an online survey program. Once on the Survey MonkeyÒ website, the first screen contained an information sheet describing the intended research as well as asking the member if he or she agrees to participate in this voluntary survey. Respondents had the option to not participate. For those participants who decided to participate, subsequent screens contained the survey questions. No identifying information was collected.

A second email was distributed two weeks after the initial invitation encouraging members to complete the survey if they had not already done so. The survey was open for only 30 days and locked out after this point. Additionally, any members of the AAST who were previously elected to receive the AAST month electronic newsletter, The Cutting Edge, were also able to view the invitation in the newsletter and participate in the study. The newsletter with the invitation was printed and distributed during the same month as the email distribution containing the survey. The data obtained in the survey were categorized using descriptive statistics and compared with the data from a poll by the New York Times (NYT) [3].

## Results

Respondent demographics

Of the AAST members canvassed, 120 completed the survey. The median age was 51. When stratifying the respondents into age groups, the majority were 49-66 years old (56%, *n *= 67), followed by those 29-48 years old (38%, *n *= 45), and lastly the eldest group, 67-88 years old (7%, *n *= 8). Most of the respondents were male (64%, *n *= 77) and worked at a Level I trauma center (80%, *n *= 96). The most common practice setting was at an academic medical center (67%, *n *= 80), followed by community medical centers (20%, *n *= 24). The majority (78%, *n *= 94) of the respondents had been in surgical practice for over 10 years, with only a minority in practice less than 10 years (22%, *n *= 26).

Gun ownership

Similar to the NYT poll (47%, *n *= 521), only below half of the responding surgeons, or someone in their household, owned a firearm (40%, *n *= 48). However, surgeons were more likely to own a gun for protection and sport, whereas the NYT survey participants were more likely to own a gun for protection and hunting. The statement that violence in popular culture is a contributing factor to gun violence in the US was agreed upon by both the NYT respondents (73%, *n *= 810) and the AAST participants (75%, *n *= 90). Similarly, 61% (*n *= 73) of the trauma surgeons and 53% (*n *= 588) of the NYT respondents were in favor of stricter gun control laws. 

Gun violence prevention

While 75% (*n *= 532) of the NYT respondents felt that having more police or security personnel in public places would decrease mass shootings, only 32% (*n *= 38) of the trauma surgeons shared this view. Forty percent of the trauma surgeons (*n *= 48) disagreed with the statement that more police presence would decrease gun violence. Eighty percent of the NYT respondents (*n *= 888) felt that mental health screening and treatment would decrease gun violence, whereas only 56% (*n *= 67) of the surgeons felt that it would be beneficial. While 90% (*n *= 999) of the NYT poll respondents were in favor of legislation restricting the sale of guns to licensed dealers, only 66% (*n *= 79) of the trauma surgeons respondents were in similar agreement. With regard to the banning of high-capacity magazines, 66% (*n *= 732) of the NYT respondents were in favor of such legislation, while the trauma surgeons were split; 44% (*n *= 53) were in favor and 44% (*n *= 53) against legislation limiting magazine capacity (Table 1) [3].

**Table 1:** NYT Data vs Trauma Surgeons NYT: New York Times

Poll Question	New York Times	Trauma Surgeons
Own or someone in household owns a gun	47%	40%
Top two reasons to own a gun	Protection, hunting	Protection, sport
Stricter gun laws would prevent more violence	53%	61%
Favor a law restricting guns sales to only licensed dealers	90%	66%
Banning high-capacity clip magazines would decrease violence	60% Favored	44% Disagreed 44% Favored
Having more police/security personnel would decrease mass shootings	75%	32%
Mental health screening would help prevent gun violence	80%	56%
Violence in popular culture is a contributing factor to gun violence in the US	75%	73%

Gun violence exposure

Trauma surgeons were queried about the number of times in their career that they provided direct patient care to more than three simultaneous firearm victims from the same incident. The majority (27%, *n *= 33) never treated three or more simultaneous firearm victim patients. Fifteen percent (*n *= 18) had one occurrence with three or more firearm victims, 18% (*n *= 22) had two occurrences, 10% (*n *= 12) had three occurrences, 4% (*n *= 5) had four occurrences, and 1% (*n* = 1) had five occurrences with three or more firearm victims. Overall, 24% (*n *= 29) of the trauma surgeons had more than five occurrences with three or more simultaneous firearm victims.

Gun violence perceptions

When ranking the potential causes of gun violence in America, trauma surgeons categorized failure to control the illegal drug trade as the highest factor. This was followed by poor parenting, gun availability, lack of adequate mental health screening and treatment, and influence from media, in decreasing order. Most agreed that improving mental health screening was the best way to decrease mass shooting incidents. The majority (64%, *n *= 77) felt that gun violence is not well studied. Eighty-seven percent (*n *= 104) acknowledged the need for more research on firearm injury and prevention.

## Discussion

The three principal findings of our study are as follows: 1) Trauma surgeons and the American populace share similar views regarding gun violence; 2) gun violence is a fiscal and burden to society; and 3) current scientific gun research is absent. The impact of popular culture, influence of police and security personnel, and the effect of mental health screening on reducing gun violence have been weakly studied, and implications made lack solid scientific evidence.

The burden of gun violence

**Figure 1:**
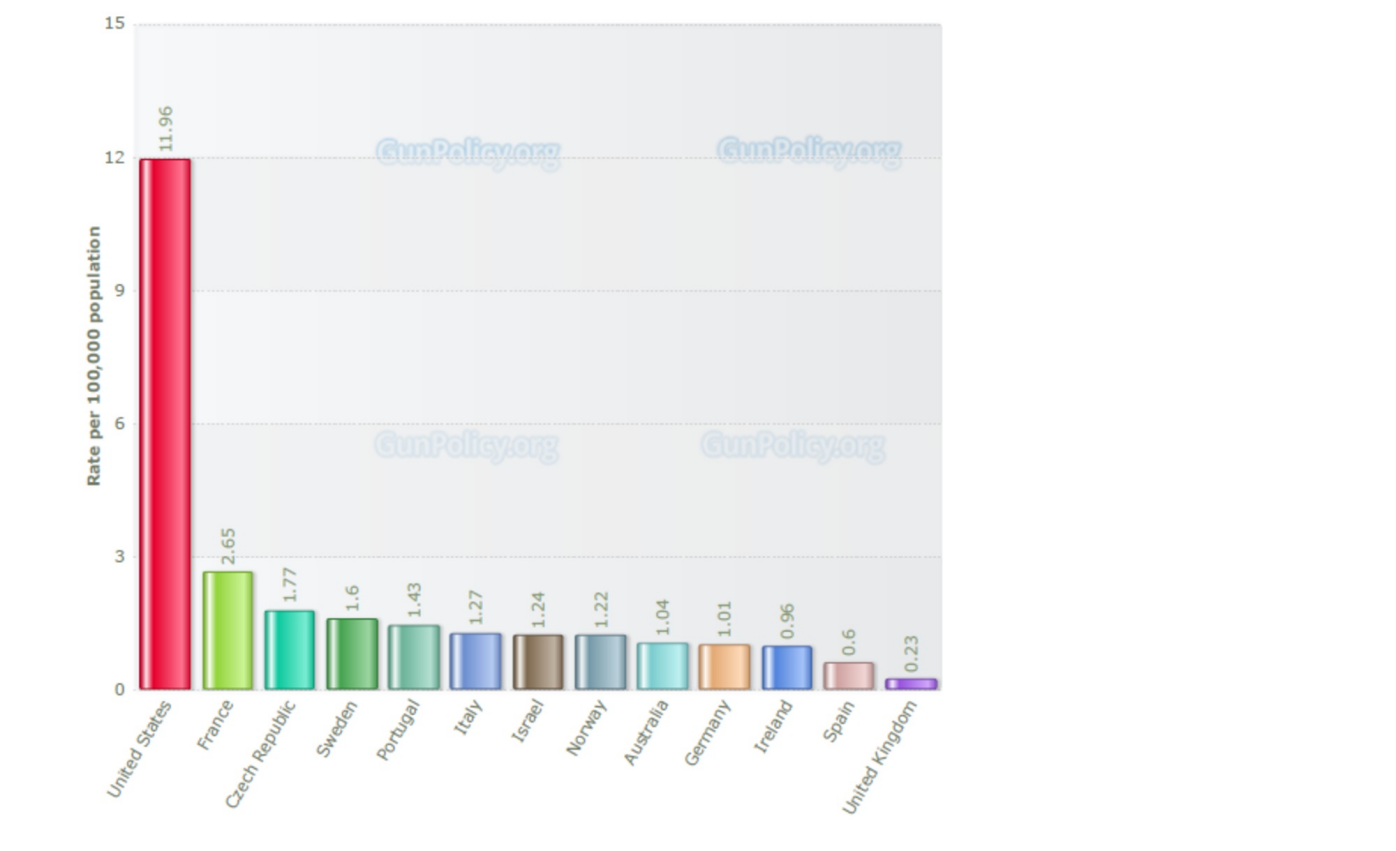
Country Gun Deaths per 100,000 People

There is significant gun violence in America, especially when compared to other nations (Figure 1) [4].

When reviewing a World Health Organization database of the 23 most populous high-income countries, the homicide rate from firearms in the US was nearly 20 times higher than that of other industrialized nations [5]. Of all combined firearm deaths in these 23 countries, the US accounted for 80% of all firearm deaths (Table 2) [5].

**Table 2:** Death Rates from Suicide and Homicide *Countries included Australia, Austria, Canada, Czech Republic, Finland, France, Germany, Hungary, Iceland, Italy, Japan, Luxembourg, Netherlands, New Zealand, Norway, Portugal, Slovakia, Spain, Sweden, United Kingdom (England and Wales), United Kingdom (Northern Ireland), and United Kingdom (Scotland).

	Death Rates in US per 100,000 population	Death Rates the Non-US High Income Countries per 100,000 population*
Overall Gun Homicide	4.1	0.2
Gun Homicide 15-24 yrs old	10.7	0.3
Overall Gun Suicide	5.8	1.0
Non-Gun Suicide	5.0	13.9
Unintentional Gun Death	0.3	0.0

The US spends nearly $100 billion annually on all associated costs of gun violence [6]. Several studies have demonstrated that the risk of suicide by firearm increases with having a gun at home by a factor of 4.8 [7].

Current firearm research (unfunded)

Little scientific research has been conducted in recent years regarding gun violence—most of the published literature is over 20 years old. Research from the 1980s and 1990s provides some information pertaining to the complex issues surrounding gun violence. In 1996, the House of Representatives defunded approximately $2.6 million in funding from the National Center for Injury Prevention and Control, subsequently dedicating the funding for traumatic brain injury research [8-9]. As a direct result of this re-appropriation, financial support for firearm injury research ceased [8-9].

Research into firearm injury prevention was further hampered when the appropriation bill added language preventing any monetary funds designated for the Centers for Disease Control to be used to “advocate or promote gun control” [10]. When future governmental agencies funded additional research regarding firearms, the Congress placed the same stipulations on funding to all Department of Health and Human Services, including the National Institute of Health [11]. Without appropriate funding and adequate government regulations to assist in the collection of information regarding the manufacturing, sale, and distribution of firearms, applicable research that can impact firearm violence is unlikely.

Gun ownership

The Second Amendment of the United States Constitution guarantees the right of an individual to bear arms. Currently, in the United States, it is estimated that 35% to 49% of the households possess a firearm. While trauma surgeons may see more victims of gun violence than the average citizen, the rates of gun ownership are similar to the general population.

The top two reasons to own a gun were different for the NYT respondents and the AAST trauma surgeons; however, both included protection as a primary reason for ownership. The NYT respondents were more likely to also choose hunting as an additional reason for ownership, while the AAST members selected sport shooting as an additional reason for gun ownership. Protection was cited as the main reason for gun ownership in both the NYT and the AAST surveys; but, does gun ownership prevent crime? The Federal Bureau of Investigation (FBI) homicide data indicates that only 385 legally justified self-defense homicides occurred in 2010 with a firearm [12]. For each time a gun was used in self-defense, there were four unintentional shootings, seven homicides or assaults, and eleven attempted or completed suicides [13]. The number of assaults, robberies, attempted murders, or other crimes that are prevented because the victim owns a firearm is unknown. The mantra for owning a gun for protection, and its actual role in preventing a crime, is not supported by evidence.

Gun control laws (legislation)

Nearly an equal percentage of the NYT respondents (53%, *n *= 588) and trauma surgeons (61%, *n *= 73) were in favor of stricter gun control laws. However, what specific type of legislation, and whether or not gun injury prevention can be obtained with legislation, remains unclear.

The handgun legislation in Vancouver, Canada is more restrictive than the handgun legislation in Seattle Washington. In 1988, Sloan and colleagues set out to compare the robbery, burglary, assault, and homicide rates in these two geographically similar cities. While both cities had similar overall rates of criminal activity and assaults, the homicide rate was seven times higher in Seattle [14]. The odds of being murdered by a handgun were four times higher in Seattle. The authors conclude that restricting access to handguns may reduce the homicide rate in a community [14].

One of the largest articles to demonstrate that strict gun control laws can decrease crime was published in 2006 from Australia. In 1996, following a heavily publicized violent gun massacre in Tasmania, a series of laws were passed to decrease gun violence in Australia. This legislation included the buy-back (at market cost) of most firearms and the mandatory registration for the few types of guns allowed [15]. It also limited all gun sales to only licensed dealers, prohibiting all personal/private sales. Nearly 700,000 weapons were turned into the police. The national crime database for Australia was reviewed 10 years after implementation. In the 18 years prior to the Australian National Firearms Agreement in 1996, there were 13 mass shootings, followed by zero since the law’s implementation [16]. The rates of firearm deaths, homicides, and suicides all decreased by substantial numbers after the law was implemented [15-16]. Firearm homicide rates decreased from 0.57 to 0.27 per 100,000 people [16]. Suicide by firearm dropped from 2.09 to 0.97 per 100,000 people [16].

It has been proposed that legislation mandating firearm education programs (like what is done for driver’s education) may decrease gun violence. Accidental shootings are estimated to account for 600 deaths and 14,675 injuries per year [17]. While (90%, *n *= 999) of the NYT poll respondents were in favor of a law restricting the sale of guns only by licensed dealers, only (66%, *n *= 79) of the trauma surgeon respondents concurred.

While the definitions of “assault weapon” and “high capacity magazine” vary, these terms are generally applied to any weapon that can allow the user to fire multiple rounds in succession without reloading. The inability to clearly define what an assault weapon is (semi-automatic, automatic, and how many rounds prior to reload…) not only impedes any type of meaningful legislation, but also compounds the difficulty of a meaningful research [18]. Nearly half of the trauma surgeons felt that placing a ban on high-capacity magazines would have no effect on gun violence. Two plausible explanations for this sentiment among the trauma surgeons include the fact that most gun violence is from small caliber pistols, and most gun violent crimes are single-victim crimes. While society recalls the gun violent crimes of mass casualties, the majority of trauma patients seen are single-incident firearm injuries. 

Impact of popular culture 

Violence in media (TV, music, games, movies) is often listed as a reason for the increasing gun violence in the United States. Nearly 75% of the AAST and NYT respondents felt that violence in popular culture is a contributing factor to gun violence in the US. In the early 1990s, it was speculated that at least part of the violence committed was contributed by the “frequent appearance of assault weapons on television” [18]. During the late 1980s and early 1990s, media productions such as "The A-Team" and "Miami Vice" and movies like Rambo “probably all had a role in increasing their [assault weapon] popularity” [18]. The toy manufacturing companies also have understood the relationship between the violence seen in media and have produced toy replicas of firearms. An estimated $40 million was spent on advertising assault weapons to similar versions of the Uzi and AK-47 [19]. 

Two articles published in 1992 and 2002 supported the notion that 10% to 30% of the violence in the society is directly attributed to the violence seen or experienced with media [20-21]. This is approximately the same impact as smoking that was banned from television advertising for its negative influence [22].

Not all research supports this association. Most researchers agree that there is a short-term effect on arousal, thoughts, and behavior, especially with boys. However, the long-term effect and the direct effect on crime is weak [23]. Most critiques of data supporting the association between violence in the media and gun violence argue the data is flawed because research does not adequately account for other significant factors predisposition for violence and aggressive behavior, prior violence experienced, and social and economic demographic data that has been linked to violence [23].

Fergurson and colleagues demonstrated that results from current analysis do not support the association between violence in the media with violence committed stating that “it cannot be concluded that media violence presents a significant public health risk” [24].

Influence of police and security personnel

While 75% (*n *= 832) of the NYT respondents felt that having more police or security personnel in public places would decrease mass shootings, only 32% (*n *= 38) of the trauma surgeons shared this view. The largest majority of trauma surgeons (40%, *n *= 48) disagreed with the statement that more police presence would decrease gun violence. Controlling all variables to definitively prove that an increase in police or security personnel will decrease violent crime is a daunting task fraught with scientific difficulty. With regard to school safety and gun violence, little national data is available. School Resource Officers (SRO), state, and local police agencies have attempted to comment on the relationship of school violence, but most data is opinion. Studies of SRO effectiveness that have measured actual safety outcomes report varying results. Some show an improvement in safety and a reduction in crime; others show no change [25].

The National Crime Prevention Council reports the SROs have been successful in communities across the country, helping to reduce violence, improving law enforcement-school relations, and enhancing positive images of law enforcement among students [26]. However, while it may be possible that a certain police force size and activity may be needed to deter crime, “increases in police numbers or intensification of traditional police activities will have no additional marginal general deterrent or crime reduction effect” [27].

Effect of mental health screening

Mass shooting disasters are often highly publicized and may even “fuel the perception that all persons with mental illness are dangerous” [28]. Current legislation in most states requires a background check prior to purchasing a handgun from an authorized dealer. This background check is under the National Instant Criminal Background Check System (NICS) and stems from the Gun Control Act of 1968 [29]. It currently restricts prohibited persons from purchasing firearms, including individuals addicted to controlled substances, those involuntarily committed to a mental institution or adjudicated as incompetent or dangerous, or those who receive a verdict of not guilty by reason of insanity [29]. The majority of mentally ill patients are not violent [28]. Research has shown that people discharged from an inpatient mental illness facility, after involuntary admission, are not any more likely to commit violence unless they have had a previous violent history or substance abuse [30].

There are several limitations to this study. Only 17% (*n *= 706) of the AAST members responded. Perhaps the respondents’ views do not correspond to the population of trauma surgeons at large. The data obtained is purely subjective and opinion from the respondents. While we assume respondents answered with sincere thought and based on life experiences, application to future projections is conjecture. Nearly all data discussed regarding gun control is difficult to apply. Also, research conducted in major metropolitan areas may not correlate to rural portions of the same state or other states. 

One conclusion that we can make is that trauma surgeons are no more well informed than the public at large due to the lack of a funding from the federal government, and the lack of a national database reporting system regarding gun purchases, tracking, and gun injuries. Injury prevention research can decrease death rates.

## Conclusions

Trauma surgeons appear to share most of the same views regarding gun violence with the public at large. Current research is needed to elucidate the optimal way to prevent future gun violence. Areas of potential research should include the influence of gun violence in popular culture, the effect of mental health screening, the impact of having only licensed dealers sell guns, the enforcement of current background checks, and most importantly, a national database for gun violence research.
